# A Novel Simulation Model for Training Emergency Medicine Residents in the Ultrasound Identification of Landmarks for Cricothyrotomy

**DOI:** 10.7759/cureus.33003

**Published:** 2022-12-27

**Authors:** Josie Acuña, Garrett Pacheco, Adrienne A Yarnish, Javier Andrade, Stephen Haight, Ian Coe, Jeremy Carter, Srikar Adhikari

**Affiliations:** 1 Emergency Medicine, University of Arizona College of Medicine, Tucson, USA; 2 Emergency Medicine, University of California, San Francisco, Fresno, USA; 3 Internal Medicine, University of Arizona College of Medicine, Tucson, USA

**Keywords:** cricothyrotomy, residents, education, airway, simulation, emergency medicine, point-of-care ultrasound, ultrasonography, ultrasound

## Abstract

Objectives

The objective of this study is to describe a simple, replicable method to create neck models for the purpose of education and practice of ultrasound (US) identification of anatomic landmarks for cricothyrotomy. The second objective is to assess the model’s capability in training emergency medicine (EM) residents in the US identification of anatomic landmarks for cricothyrotomy.

Methods

This is a cross-sectional study using a convenience sample of EM residents. Participants were taught to identify the thyroid cartilage, the cricothyroid membrane (CTM), and the cricoid cartilage using US. After an instructional period, participants performed a US examination on gel models designed to overly a live, human neck simulating various scenarios: thin neck, thick neck, anterior neck hematoma, and subcutaneous emphysema. Residents were asked to identify the thyroid cartilage, the CTM, and the cricoid cartilage as quickly as possible. The mean time to successful identification was reported in seconds. Following the scanning session, participants were asked to complete a post-survey. After the session, the video recordings were reviewed by an emergency US fellowship-trained physician to assess the visuomotor skills of each participant.

Results

A total of 42 residents participated in the study. Ninety-three percent (32/42; 95% CI 80.3% - 98.2%) of residents were able to obtain an optimal sagittal or parasagittal sonographic view of the anterior airway landmarks. Of these residents, 21.4% (9/42; 95% CI 11.5% - 36.2%) required minimal assistance with the initial probe placement. The visuomotor scores were recorded for each participant. Results of the pearson correlation indicated that there was a significant positive relationship between the residents’ year in training with their visuomotor score (r(40) = .41, p = .007). When scanning the thin neck, 90.5% (38/42; 95% CI 77.4% - 96.8%) of residents were able to successfully identify the landmarks. The median time to completion was 27 seconds.

When scanning the subcutaneous air model, 88.1% (37/42; 95% CI 74.5% - 95.3%) of residents were able to successfully identify the landmarks. The median time to completion was 26 seconds. When scanning the neck with the fluid collection 95.2% (40/42; 95% CI 83.4% - 99.5%) of residents were able to successfully identify the landmarks with a median time of 20 seconds for identification. When scanning the thick neck model, 73.8% (31/42; 95% CI 58.8% - 84.8%) of residents were able to successfully identify the landmarks taking a median time of 26 seconds. After the training session, 76.2% of residents reported that they felt either “confident” or “extremely confident” in identifying the CTM using US.

Conclusion

The novel anterior neck gel models used in this study were found to be adequate for training EM residents in the US identification of anterior neck anatomy. Residents were successfully trained in identifying the important anterior neck landmarks that are useful when predicting a difficult anterior airway and planning for surgical cricothyrotomy. Residents overall felt that the models simulated the appropriate anatomic scenarios. The majority felt confident in identifying the CTM using US.

## Introduction

Cricothyrotomy in airway management is the definitive intervention when confronted with the “cannot intubate, cannot ventilate” scenario [1]. The success of this procedure relies on the accurate identification of the cricothyroid membrane (CTM). The inability to identify the CTM by external visualization or palpation is an important contributor to decreased success in this procedure, causing damage to local structures, airway injury, and misplacement [2]. In order to improve the success rate of cricothyrotomy, it has been recommended to identify and mark the CTM before the management of the anticipated anatomically difficult airway [3]. Unfortunately, prior studies have demonstrated poor accuracy in identifying the CTM with conventional palpation techniques among certain groups, such as female patients compared to males, obese patients, or those with distorted neck anatomy [4,5]. Cricothyrotomy is a high-acuity, low-opportunity procedure; therefore, emergency medicine (EM) trainees must obtain experience in evaluating these types of patients prior to performing cricothyrotomy. Ultrasound (US) has been found to be superior compared to palpation to accurately identify significant anatomic landmarks of the anterior neck [6-8], making it a potentially useful tool when predicting a difficult anterior airway and planning for cricothyrotomy. However, the utility and accuracy of US is highly dependent on the ability of the operator to acquire images of adequate diagnostic quality, highlighting a need for adequate training in EM residency. Due to the need for experience in patient evaluation and concurrent effective use of US, it is critical that the simulation of US identification of CTM plays a large role in resident training. Numerous studies have shown that procedural ultrasonography can be taught through simulation [9-12]. However, to our knowledge, there are no commercially available simulation models to train EM physicians in the US evaluation of the anterior neck when preparing for surgical cricothyrotomy. The objective of this study is to describe a simple, replicable simulation model of the anterior neck, modifying the anatomy of a live human neck model, for the purpose of education and practice of US identification of anatomic landmarks for cricothyrotomy. The second objective is to assess the model’s capability in training EM residents in the US identification of anatomic landmarks for cricothyrotomy.

This article was previously presented at the American Institute of Ultrasound in Medicine (AIUM) Annual Meeting on March 12, 2022, and the Society for Academic Emergency Medicine (SAEM) Annual Meeting on May 11, 2022.

## Materials and methods

This is a cross-sectional study using a convenience sample of residents. The study was approved by the Institutional Review Board at the University of Arizona. Informed consent was obtained from all subjects. This study was conducted at two academic medical centers with two Accreditation Council for Graduate Medical Education-accredited categorical three-year EM residency programs and a five-year combined EM and pediatric residency program. There is a robust US training program for residents including an emergency US fellowship program. Residents are required to do a two-week rotation in emergency US during their first year in training, and they are also encouraged to perform US examinations under faculty supervision while on shift. The data were collected from residents training at one of the three residency programs.

**Figure 1:**
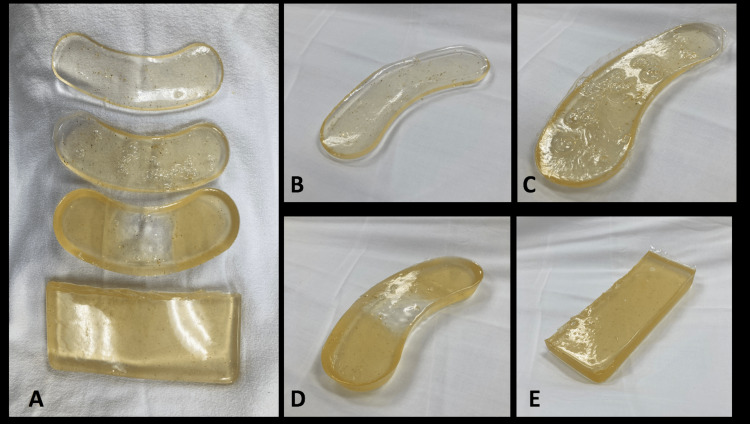
Representative images of (A) anterior neck gel models, (B) normal, thin anterior neck model, (C) neck model with simulated subcutaneous air, (D) model simulating hematoma or fluid collection, (E) thick, obese neck model

Data were collected on each participant’s level of training and their estimated number of US studies performed prior to evaluation. One week prior to the data collection portion of the study, participants were given reading materials to review on performing the US of the anterior neck and a review of relevant anatomy. Just prior to performing scans on the simulation models, participants received a short, hands-on didactic session with the opportunity to scan the neck of a human model. Participants were taught to identify the thyroid cartilage, the CTM, and the cricoid cartilage in a sagittal plane. After the instruction period, participants performed the same US examination on the gel models designed to overly a live, human neck simulating various scenarios. The human neck model was female with a normal body mass index. Models were made from an inexpensive, commercially available gel mold, Composimold © made of a thermoplastic resin. A total of four models were made that simulated various anterior neck anatomy. The four anterior neck models used are shown in Figure 1 and were as follows: (1) normal thin anterior neck model, (2) neck model simulating subcutaneous air, (3) simulated anterior hematoma or fluid collection over the anterior neck, and a (4) thick, obese neck model. On the live neck model, the distance from the skin to the CTM was 1 cm. The thickness of a thin neck simulation model that was made to overly the live model was 0.75 cm and the thick neck simulation model was 2.0 cm. To create the hematoma/fluid collection model, saline encased in a small plastic lining was inserted into the gel mold as it was setting. To create the subcutaneous air model, the air was injected into the gel mold to create several pockets.

Participants scanned each of the gel models overlying the human model in the stated order. They were asked to identify the thyroid cartilage, the CTM, and the cricoid cartilage as quickly as possible. The session was video recorded for quality control. This external video recording was combined with a concurrent recording of the US images. The participant’s face and any identifiers were not visible on the recording. A Mindray M9 (Mahwah, NJ, USA) cart-based US machine was used for this study. A high-frequency linear probe was used for all examinations. Times for placement of the probe on the gel to time to identification of the structures were recorded in real-time and later confirmed by video recording. Residents were given one attempt to identify the structures using each model. The maximum time allowed for each attempt to identify all structures was 60 seconds, and a single attempt was allowed on each model. Correct identification was confirmed by an instructor. The difference in the proportion of successful CTM identification was reported with 95% confidence intervals (CIs). The mean time to successful identification was reported in seconds. Following the scanning session, participants were asked to complete a post-survey. After the session, the video recordings were reviewed by an emergency US fellowship-trained physician to assess the visuomotor skills of each participant. Each participant’s attempts were reviewed for verbal prompting by the instructor and the need for the instructor to adjust the participant’s probe position. Following a single viewing of video recordings, the reviewer also provided an overall assessment of each participant’s perceived visuomotor skills using a scale of 1 through 5 (1 = Poor, 5 = Strong). Scoring was based on whether the residents made appropriate adjustments with the probe to enhance their image, and obtained final images that were of adequate quality for interpretation. The application of adequate pressure was also a component of this evaluation. It was reviewed whether there was poor probe contact with the model, or conversely whether the probe was seen to cause significant protrusion into the gel model as seen on the machine display.

## Results

**Figure 2:**
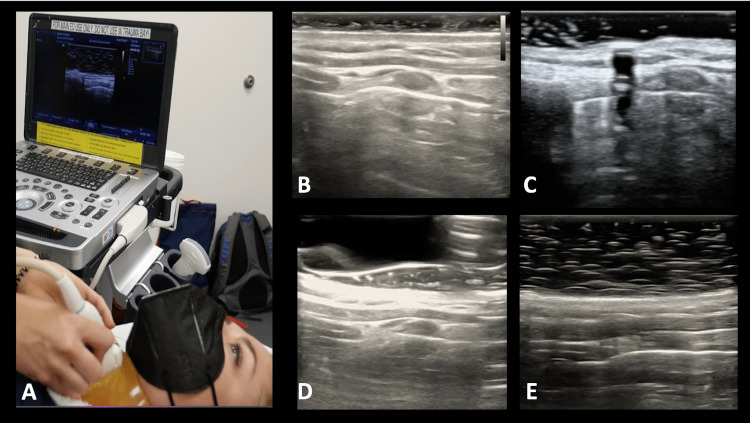
Representative images of (A) examination set-up with gel model placement over the anterior neck, (B) ultrasound of normal thin anterior neck model, (C) ultrasound of neck model with subcutaneous air, (D) ultrasound of model with hematoma or fluid collection over the anterior neck, (E) ultrasound of thick, obese neck model

**Figure 3:**
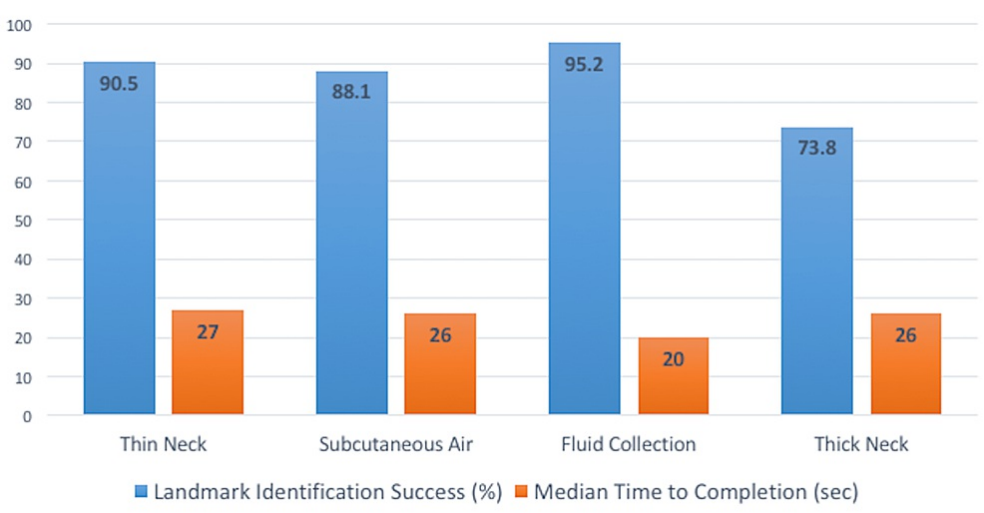
Percentage of successful identification of anterior neck landmarks on ultrasound by residents and median time to completion

A total of 42 residents participated in the study. They were all at various levels in their training (Table 1). Prior to their participation in this study, they had performed a various number of point-of-care US examinations during their training, ranging from less than 50 to over 200 examinations (Table 2). Ninety-three percent (32/42; 95% CI 80.3% - 98.2%) of residents were able to obtain an optimal sagittal or parasagittal sonographic view of the anterior airway landmarks (Figure 2). Of these residents, 21.4% (9/42; 95% CI 11.5% - 36.2%) required minimal assistance with the initial probe placement. The visuomotor scores were recorded for each participant (Table 3). Results of the pearson correlation indicated that there was a significant positive relationship between the residents’ year in training with their visuomotor score (r(40) = .41, p = .007). The results for the successful identification of the landmark structures and median time to completion are demonstrated in Figure 3.

**Table 1:** Resident levels of training by year

Level of training	Number of residents
Post-graduate year 1	16
Post-graduate year 2	12
Post-graduate year 3	14

**Table 2:** Resident estimated number of ultrasound examinations performed during residency training

Estimated number of ultrasound examinations performed during residency training	Number of residents
<50	2
50-100	6
100-150	10
150-200	9
Greater than 200	15

**Table 3:** Resident visuomotor scores

Visuomotor score	Post graduate year	Number of residents
1	1	3
	2	0
	3	0
2	1	3
	2	5
	3	1
3	1	6
	2	3
	3	2
4	1	0
	2	3
	3	4
5	1	4
	2	1
	3	6

After the training session, residents were asked to gauge their level of confidence in interpreting the images they obtained using the various models, using a scale of 1 to 10 (1 = low confidence 10 = high confidence). The mean confidence level for the thin neck models was 8.4 (SD ± 2.1), 6.6 (SD ± 2.4) for the subcutaneous air model, 7.6 (SD ± 2.2) for the fluid collection model, and 7.4 (SD ± 2.2) for the thick neck model. Residents were also surveyed on their perception of the various gel models. They were asked to what extent they agreed that the gel models simulated the appropriate anatomic scenario (thin neck, subcutaneous air, etc.) using a scale from 1 through 10 (1 = strongly disagree, 10 = strongly agree). For the thin neck model, the mean score was 5.3 (SD ± 3.9), for the subcutaneous air model, 5.4 (SD ± 3.5), for the fluid collection model, 5.3 (SD ± 3.6), and for the thick neck model, 5.6 (SD ± 3.4). After the training session, 76.2% of residents reported that they felt either “confident” or “extremely confident” in identifying the CTM using US.

## Discussion

Data from the Fourth National Audit Project 4 of the Royal College of Anesthetists have suggested that lack of planning is a contributor to poor airway outcomes, particularly, failure to plan for failure [13]. The declaration of a failed airway and a "cannot intubate, cannot ventilate" situation should prompt physicians to perform a surgical cricothyrotomy. Practice guidelines for difficult airway management recommend that the operator prepares a strategy that may conclude with securing a patent airway via the anterior neck [3,14,15]. Inexperience and inability to identify the CTM by palpation are common contributors to failure. Therefore, with recognition of the anatomically difficult airway, it is critical to accurately identify the CTM prior to the initial airway attempt should the situation lead to cricothyrotomy. One tool to achieve accurate identification of the CTM is through the use of US. With accurate use of US, the CTM can be appropriately identified as part of the airway strategy prior to the onset of the procedure. Since clinical opportunities for EM trainees to practice such skills are relatively infrequent, training for these high-acuity, low-opportunity situations relies on simulation. In this study, we introduced novel simulation models of the anterior neck. These models were used in the training of residents to use US to identify the landmarks of the anterior neck necessary for planning for surgical cricothyrotomy.

Our study shows a favorable outcome with regard to the usability of novel simulation gel models of the anterior neck for practice and education. Residents found the models to be adequate in demonstrating the various clinical scenarios of the normal anterior neck; the neck with subcutaneous air, the presence of an anterior hematoma or fluid collection over the anterior neck, and the thick, obese neck model. Identification of the thin neck was much more successful in the present study than in previous research conducted on human models. Our participants had a 90.5% success rate (38/42; 95% CI 74.5% - 95.3%) with identifying the thin neck, as opposed to a study on healthy non-obese volunteers comparing EM residents using US (69.2%) and landmark techniques for identification of the CTM (66.7%) [16]. In our study, residents were able to obtain an adequate view of the CTM within a median time of 27 seconds, whereas the comparative study found that US led to a prolonged time for localization as compared to palpation (17 ± 9.2 seconds vs 8.25 ± 4.8 seconds, respectively). We did not directly compare time to identification with US compared to conventional palpation techniques, but 27 seconds is a seemingly rapid time to identify important clinical landmarks, supporting the utility of US identification of the CTM in thin anterior neck patients.

Success rates in the present study decreased in some conditions that classically render the CTM more difficult to identify, but remained higher than in standard palpation technique [5]. When the anatomy was distorted with subcutaneous air to mimic airway injury, residents’ success was still 88.1% (37/42; 95% CI 74.5% - 95.3%), and with the thick, obese neck model, their success decreased to 73.8% (31/42; 95% CI 58.8% - 84.8%). To our knowledge, this is the first study using a neck model with subcutaneous air for US identification of the CTM. Air may lead to poor image quality when using US and may have been the reason for decreased identification success. The thick, obese neck model also demonstrated decreased success with residents being able to identify the CTM 73.8% (31/42; 95% CI 58.8% - 84.8%). It is more likely that the additional difficulty with the thick, obese model is secondary to increased tissue thickness. On the other hand, when scanning the neck with the fluid collection, EM residents were able to identify the CTM 95.2% (40/42; 95% CI 83.4% - 99.5%) successfully. This was likely secondary to the fluid being an ideal medium to improve US image quality. Although we did not directly compare it to conventional palpation, current literature suggests that US leads to higher identification success compared to conventional palpation in obese patients [5]. Furthermore, US has also been shown to increase identification success with abnormal neck anatomy [17]. Thus, our results for our thick/obese and distorted anterior neck models are comparable. The median times to identification were comparable between all of the different neck models used in our study. Unfortunately, this may have been due to the learning effect of each US operator performing the procedures in order. Our findings suggest that the neck models we developed are adequate to be used for simulation training in the identification of the anterior neck landmarks necessary for performing a cricothyrotomy.

An additional objective of this study was to evaluate the structural and functional fidelity of this novel task trainer and assess its capability for training EM residents in the US identification of anatomic landmarks for cricothyrotomy. Commercially available simulation models are usually based on the anatomy of thin adults without pathology. Previous studies that report training using human cadavers have described the exclusion of specimens that were obese or had neck pathology [18-20]. Previous publications have described novel manikins for surgical cricothyrotomy, but these have not been paired with US identification. We developed multiple anterior neck models to mimic pathology that are more consistent with those observed in real clinical situations. Our results demonstrate that the different models are feasible simulators to better prepare EM residents for this rare procedure.

One component of this study was to assess the residents’ visuomotor skills when performing a US examination on the models, referred to more commonly as hand-eye coordination. When a US examination is performed, fundamental visuomotor skills are used to enable an operator to move and manipulate a transducer in response to sensory information [21]. Visuomotor skills evolve from the sonographer having an initial mental image of what the US image should look like for a given clinical scenario [22,23]. The majority of the residents scored at least 3 out of 5 on the visuomotor scale when scanning the models, demonstrating at least average skills. Notably, the only residents who scored a 1 out of 5, i.e. poor visuomotor skills were post-graduate year 1 residents. Visuomotor skills are central components of US education and should be evaluated for the purpose of improving training and assessment. Unfortunately, assessments of these skills when performing procedures have always been difficult to assess. As of yet, there is no standard to measure US visuomotor skills in a manner that is objective and reproducible between instructors.

This study has several limitations including the controlled environment of the simulation laboratory may not be reflective of an actual clinical scenario with a real patient. As with all studies involving the psychomotor performance of technical skills, generalizing results to real patients may be difficult. Additionally, the gel models may lack actual soft tissue characteristics, rendering it a less realistic surrogate of a patient’s neck. There are limitations with regard to operators and procedures as well. As in any study of this nature, operators may have been acting in a way that does not accurately reflect the manner in which they would perform US in real patients. For example, the knowledge that the study session was conducted in a simulation environment may have led to a decreased emphasis on proper technique and urgency. However, each operator received the same hands-on didactic session prior to the procedure, and instruction to treat the scenario as real and perform the US identification as they would with a real critically ill patient. We believe that each operator acted as their own control when comparing the US identification of each neck model, thus minimizing potential bias from these factors. As participants, EM residents may have less experience with anterior neck anatomy and the cricothyrotomy procedure in general, potentially limiting the ability to generalize the findings to other clinical settings. However, all of the EM trainees received similar airway didactics and rigorous laboratory simulation experience.

Another limitation is the small sample size, which may limit the study’s generalizability and the ability to identify small differences in performance. We also used a convenience sample of residents, which might have introduced selection bias. We also did not randomize the order of the identification for the various neck models. This may account for the shorter time to successful identification in the models which were presumed to be more difficult models. There may have been learning effects that have influenced our results. In an effort to make a comprehensive assessment of the EM residents’ training in the US identification of anatomic landmarks for cricothyrotomy, a visuomotor score was obtained. Unfortunately, there is no standardized, objective, quantitative method for assessing visuomotor skills in US. Further research would benefit from focusing on the development and validation of tools for assessing visuomotor skills in US.

## Conclusions

The novel anterior neck gel models used in this study were found to be adequate for training EM residents in the US identification of anterior neck anatomy. Residents were successfully trained in identifying the important anterior neck landmarks that are useful when predicting a difficult anterior airway and planning for surgical cricothyrotomy. After the training session, the vast majority of the residents felt confident in identifying the CTM using US.
